# PD-1 inhibitors plus lenvatinib versus PD-1 inhibitors plus regorafenib in patients with advanced hepatocellular carcinoma after failure of sorafenib

**DOI:** 10.3389/fonc.2022.958869

**Published:** 2022-09-13

**Authors:** Yongkang Xu, Shumin Fu, Kai Shang, Jiayu Zeng, Ye Mao

**Affiliations:** ^1^ Department of Oncology, The Second Affiliated Hospital of Nanchang University, Nanchang, China; ^2^ Department of Oncology, The First Affiliated Hospital of Nanchang University, Nanchang, China; ^3^ School of Clinical Medicine, Guizhou Medical University, Guiyang, China

**Keywords:** PD-1 inhibitors, lenvatinib, regorafenib, sorafenib, hepatocellular carcinoma

## Abstract

**Background:**

Lenvatinib, regorafenib and anti-programmed cell death protein-1 (PD-1) immunotherapy have shown promising clinical outcomes in patients with advanced hepatocellular carcinoma (HCC) after sorafenib failure, respectively. However, the combination of the two treatments has not been reported. We compared the efficacy of PD-1 inhibitors with lenvatinib (PL) and PD-1 inhibitors plus regorafenib (PR) in patients with advanced HCC in this study.

**Methods:**

We conducted a retrospective study of advanced HCC patients who undergone PD-1 inhibitors combined with lenvatinib or regorafenib after failure of sorafenib at Second Affiliated Hospital of Nanchang University from July 2018 and December 2020. The overall survival (OS), progression-free survival (PFS), effective rates and treatment-related adverse events (TRAEs) were investigated.

**Results:**

In total, 61 patients met the criteria and were included in the present study, and they were divided into the PL group (n = 32) and PR group (n = 29). The overall response rate (ORR) (12.5%vs. 10.3%, respectively; p = 0.557) and disease control rate (DCR) (71.9%vs. 58.6%, respectively; p < 0.207) were higher in the PL group than in the PR group, but there was no statistical difference.

Furthermore, median PFS and OS were not significantly different between the two groups in Kaplan-Meier survival analysis (PFS: 5.3 months vs 4.0 months, p = 0.512; OS: 14.1 months vs 13.7 months, p = 0.764 for the PL group vs PR group). The most common treatment-related adverse events (TRAEs) were hand -foot skin reaction (24/61,39.3%), hypertension (20/61,32.8%) and hypothyroidism (13/61,21.3%). The frequent TRAEs (≥Grade 3) during PD-1 inhibitors plus lenvatinib or regorafenib treatment were hand-foot skin reaction (5/29,12.4%), thrombocytopenia (2/29 6.90%) and proteinuria (n =2/32,6.25%).

**Conclusions:**

Combination of lenvatinib/regorafenib and PD-1 inhibitors is a promising therapy for HCC patients after sorafenib failure.

## Introduction

In 2020, the incidence of Hepatocellular carcinoma (HCC) in the world ranked sixth among malignant tumors, and the mortality rate was the third in malignant tumors (1).Most patients are already at the advanced stage at the time of diagnosis and are thus not candidates for surgical treatment due to its insidious onset (2),systemic therapy is a only potentially treatment option for patients with advanced HCC (3, 4).The SHARP trial and Asia-Pacific trial (5, 6)was shown that sorafenib can prolong overall survival over placebo and has become the standard therapy for advanced HCC. Due to its low response rate and relatively high toxicity, many patients eventually develop progressive disease or intolerance to drug. Systemic therapy for advanced HCC has changed drastically in recent years. In the phase 3 trial on regorafenib, the median PFS was 3.1 months in the sorafenib group and 1.5 months in the placebo group (P < 0.001) and the median OS was 26.0 months (7). REFLECT (8)study demonstrated that lenvatinib was non-inferior to sorafenib in overall survival in advanced HCC (median survival time: 13.6 months for lenvatinib vs. 19.6 months for sorafenib). According to the National Comprehensive Cancer Network (NCCN) Guidelines for Hepatobiliary Cancers, second-line treatment with lenvatinib or regorafenib is a valid option for advanced HCC after sorafenib failure. The emergence of immunotherapy has dramatically changed the landscape of HCC treatment. Based on the CheckMate 040 study (9) and KEYNOTE-224 study (10)show that Nivolumab and Pembrolizumab have definite curative effect on liver cancer after sorafenib treatment. With the publication of IMbrave150 (11) and KEYNOTE-524 (12)research results, immune therapy combined targeted therapy has gradually become a new treatment option. At the same time, it also brings new methods and new ideas to the second-line treatment of advanced HCC.

There are currently few data on immune checkpoint inhibitors (ICIs) combined with molecular targeted agent (MTA) in the second-line treatment of advanced HCC, therefore, we analyzed the efficacy and safety of the patients who received PD-1 inhibitors combined with lenvatinib (PL) versus those who received PD-1 inhibitors plus regorafenib (PR) after sorafenib resistance.

## Methods

### Patients

We recruited HCC patients who underwent PD-1 inhibitors combined with lenvatinib or regorafenib through July 2018 and December 2020 at the Department of Oncology, the Second Affiliated Hospital of Nanchang University. Inclusion criteria: (1)Patients were diagnosed with imaging or pathology [AASLD guidelines (13)] (2)Barcelona Clinic Liver Cancer stage B/C, or BCLC B who are not suitable for surgical treatment.(3)HCC patients who were receiving PD-1 inhibitors combined with lenvatinib or regorafenib after sorafenib failure;(4)ECOG PS (Performance score of Eastern Cooperative Oncology Group)0-2 score(5)Child-Pugh (CP) was classified as A/B(6)Patients had at least two cycles of PD-1 inhibitors plus lenvatinib or regorafenib.

The excluded criteria included concurrent with other malignancies and severe heart, lung, liver and kidney failure. The study protocol was approved by the ethics committee of the Second Affiliated Hospital of Nanchang University.

### Treatment procedures

Six available PD-1 inhibitors including camrelizumab, pembrolizumab, nivolumab, sintilimab, toripalimab, tislelizumab were prescribed intravenously. The patients were treated with camrelizumab at a fixed dosage of 200 mg every 3 weeks, pembrolizumab at a fixed dosage of 200 mg every 3 weeks, nivolumab at a dosage of 3.0 mg/kg every 2 weeks, sintilimab at a fixed dosage of 200 mg every 3 weeks, toripalimab at a fixed dosage of 240 mg every 3 weeks, tislelizumab at a fixed dosage of 200 mg every 3 weeks.

Patients received lenvatinib orally at the dose of 12 mg (body weight ≥60 kg) or 8 mg (body weight <60 kg) once daily. Patients received regorafenib orally at the dose of 120 mg once daily,3 weeks of work on and 1 week off. The lenvatinib dose could be reduced to 8 mg or 4 mg/day or further to 4 mg every other day if necessary to manage toxicity and adverse events.The dose of regorafenib can be adjusted by 40mg (40mg-80mg/day) or temporarily interrupted (less than 7 days).The decision to treat patients with TACE is based on the discussions of the multidisciplinary tumor board. Treatment with the progression of tumor, deterioration of liver function, intolerable adverse events or death can be stopped. Blood routine, liver and kidney function, urine routine, tumor markers, thyroid function, myocardial zymogram and other related laboratory tests were examined each time. To evaluate tumor response from treatment, contrast-enhanced CT/MRI was performed every 4-8 weeks.

### Efficacy and safety assessments

The treatment efficacy was evaluated according to RECIST1.1 standard (14). Objective response rate (ORR) was defined as the sum of complete response (CR)and partial response (PR), and disease control rate (DCR)was defined as the sum of as the sum of the rates of CR, PR, and stable disease (SD). Progression-free survival (PFS) was defined as the interval between the first day of treatment and the date of tumor progression or patient death from any cause. overall survival (OS) was calculated from the date that combination therapy was initiated and the date of death, or the last day of follow-up. Adverse drug reactions were evaluated by the National Cancer Institute General toxicity criteria (NCI-CTC 5.0).

### Statistical analysis

Patients were followed until death or the last follow-up date (1 October 2021). Continuous variables are expressed as medians and ranges, and categorical variables are expressed as numbers or frequencies. Continuous variables were analyzed using Student’s t-test. Categorical variables were analyzed using the chi-square test. PFS and OS were calculated using the Kaplan-Meier method, with log-rank test. The survival curve was obtained by running R version 3.6.3. Version. All statistical analyses were performed using he statistical software SPSS 26.0 software (IBM corporation). The factors with P<0.05 in univariate analysis were further merged into the Cox proportional hazards regression model to determine the factors independently related to PFS and OS. P<0.05 was considered statistically significant.

## Results

### Patients

**Figure 1:**
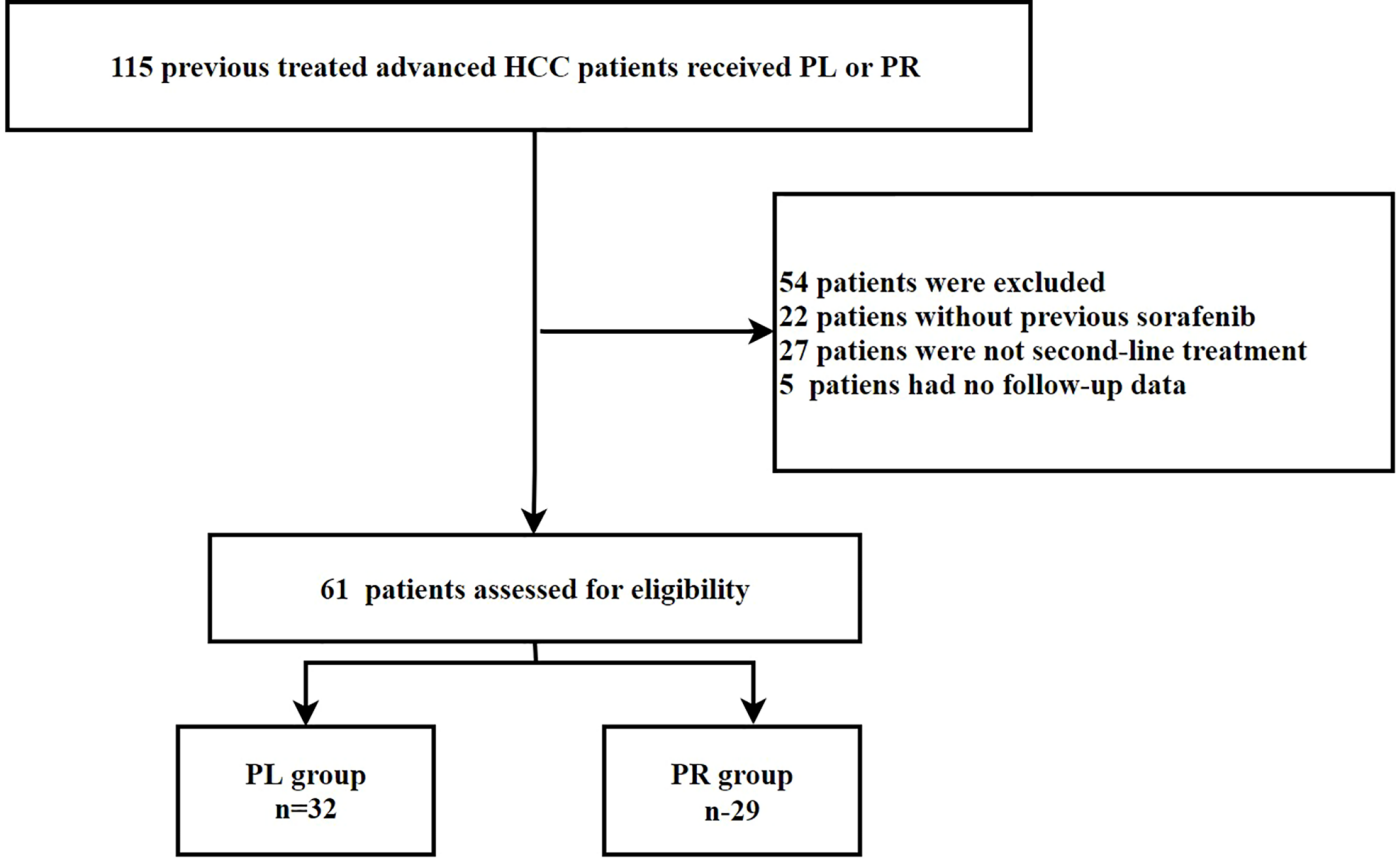
Flow chart of patient selection.

A total of 115 patients with advanced HCC who received ICIs plus lenvatinib or regorafenib were eligible. Among them, 54 patients were excluded: 22 because they did not receive sorafenib, 27 because they were not second line, and 5 because they had no follow-up data. Thus, 32 PD-1 inhibitors + lenvatinib -treated (PL group) HCC patients and 29 PD-1 inhibitors + regorafenib -treated (PR group) patients were analyzed. The patient selection process is shown in **Figure 1**.

The median age of patients was 51 (range, 17–80 years), with 39 patients (55.7%) aged above 50. Of all patients, 55 (90.2%) were male and 6 (9.8%) were female (29 males in the PL group and 26 males in the PR group). Twenty-four patients (39.3%) had an ECOG performance status ≥2 (37.5% vs. 41.4%) at the beginning of the treatment, The patients’ Child–Pugh scores was A (n = 52), with 81.3% in the PL group and 89.7% in the PR group, the patients’ ALBI scores were 1 (n = 19), 2 (n = 38), and 3 (n = 4). The aetiologies of HCC were hepatitis B virus (HBV) in 50 patients (82.0%) and Non-HBV/Non-HCV (hepatitis C virus) in 11 patients (18.0%). Among the patients, 19 patients (59.4%) in PL group and 15 patients (51.7%) in PR group received TACE therapy, respectively. The characteristics of patients are shown in **Table 1**. The ICIs type in each group are summarized in **Table 2**.

**Table 1:** Characteristics of patients in this study.

Characteristics n (%)	PL ( n = 32 )	PR ( n = 29 )	P-value
Age			0.319
≥50	21 (65.6)	14 (48.3)	
<50	11 (34.4)	15 (51.7)	
Gender			0.616
Male	29 (90.6)	26 (89.7)	
Female	3 (9.4)	3 (10.3)	
ECOG PS			0.797
0	9 (28.1)	6 (20.7)	
1	11 (34.4)	11 (37.9)	
2	12 (37.5)	12 (41.4)	
Child-Pugh			0.289
A	26 (81.3)	26 (89.7)	
B	6 (18.8)	3 (10.3)	
ALBI			0.09
1	7 (21.9)	12 (41.4)	
2	24 (75.0)	14 (48.3)	
3	1 (3.1)	3 (10.3)	
BCLC			0.562
B	9 (28.1)	6 (20.7)	
C	23 (71.9)	23 (79.3)	
Aetiology of HCC			0.199
HBV	28 (87.5)	22 (75.9)	
Non-HBV, non-HCV	4 (12.5)	7 (24.1)	
Liver cirrhosis			0.130
YES	27 (84.4)	20 (69)	
NO	5 (15.6)	9 (31)	
Portal vein tumor thrombus			0.183
YES	11 (34.4)	6 (20.7)	
NO	21 (65.6)	23 (79.3)	
Extrahepatic spread			0.123
YES	20 (62.5)	23 (79.3)	
NO	12 (37.5)	6 (20.7)	
Combination of TACE			0.366
YES	19 (59.4)	15 (51.7)	
NO	13 (40.6)	14 (48.3)	
Baseline AFP ng/mL			0.439
≥400	10 (31.3)	13 (44.8)	
<400	22 (68.7)	16 (55.2)	
Median treatment duration of sorafenib	4.7 (3.5-6.0)	3.9 (1.0-4.5)	0.156

PL, PD-1 inhibitors plus lenvatinib; PR, PD-1 inhibitors plus regorafenib;BCLC, Barcelona Clinic Liver Cancer; ECOG PS, Eastern Cooperative Oncology Group Performance status; HBV, hepatitis B virus; HCV, hepatitis C virus; AFP, alpha-fetoprotein; HCC, hepatocellular carcinoma; TACE, transarterial chemoembolization; ALBI, albumin-bilirubin.

**Table 2:** The type of immune checkpoint inhibitors(ICIs).

Drug	PL group n (%)	PR group n (%)
Camrelizumab	11 (34.38)	15 (51.72)
Pembrolizumab	1 (3.13)	0
Nivolumab	1 (3.13)	0
Sintilimab	12 (37.5)	7 (24.14)
Toripalimab	7 (21.88)	5 (17.24)
Tislelizumab	0	2 (6.90)

At the deadline of the study, 4 patients in PL group continued the original regimen, and 7 patients received third-line treatment, including hepatic arterial infusion chemotherapy (HAIC) combined with PD-1 inhibitors and apatinib(2), PD-1 inhibitors combined with regorafenib (2), regorafenib (2) and apatinib (1). One patient received radiotherapy. 12 patients received the best supportive treatment. In the PR group, one patient continued the original regimen at the deadline. After the end of the study, and 5 patients received the third -line treatment, including HAIC plus lenvatinib (2), HAIC combined with PD-1 inhibitors and apatinib (1), apatinib (1).10 patients received the best supportive treatment.

### Treatment outcomes and survival outcome

**Figure 2:**
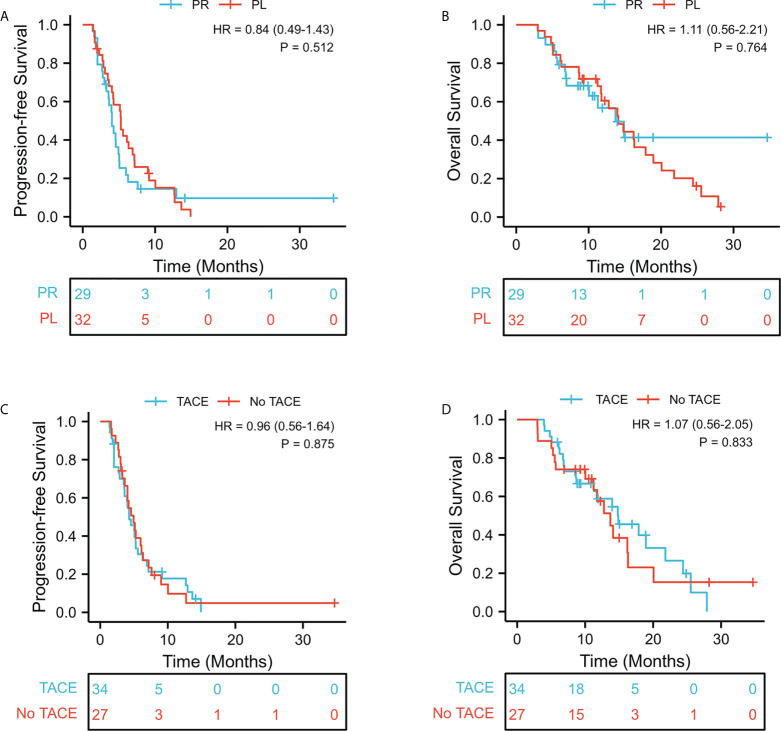
Kaplan-Meier curves of survival outcomes of patients in the two groups. **(A)** Progression-free survival. **(B)** Overall survival. Kaplan-Meier curves of survival outcomes of patients in the TACE/No TACE groups. **(C)** Progression-free survival. **(D)** Overall survival.

**Figure 3:**
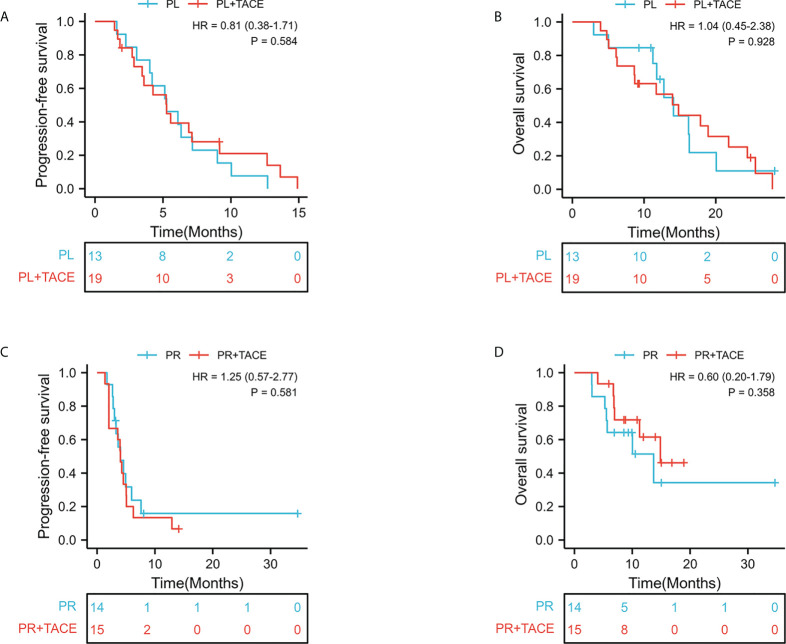
Kaplan-Meier curves of survival outcomes of patients in the PL groups. **(A)** Progression-free survival. **(B)** Overall survival. Kaplan-Meier curves of survival outcomes of patients in the PR groups. **(C)** Progression-free survival. **(D)** Overall survival.

According to Response Evaluation Criteria in Solid Tumor version 1.1, as shown in **Table 3**. In the PL group, one patient (1/32,3.1%) achieved CR, 9.4% (3/32) had PR, 59.4% (19/32) maintained SD, and 28.1% (9/32) had PD. In the PR group, 0% of patients achieved CR, 10.3% (3/29) had PR, 48.28% (14/29) maintained SD, and 41.38% (12/29) had PD. The ORR and DCR were significantly higher in the PL group than those in the PR group (12.5% versus 10.3%, P = 0.557; 71.9% versus 58.6%, P = 0.207, respectively). However, there was no statistical difference. The median follow-up period was 10.8 months. The median PFS in the PL group was 5.3 months (95% CI, 4.67–5.85) compared with 4.0 months (95% CI, 3.23–4.84) in the PR group (HR = 0.84, 95% CI 0.49–1.43; p = 0.512; **Figure 2A**). The median OS was 14.1 months in the PL group and 13.7 months in the PR group (HR = 1.11; 95% CI 0.56–2.21; p =0.764; **Figure 2B**). After stratification by absence or presence of TACE, the median PFS was 4.2 months (95% CI, 3.14–5.52) in the presence of TACE group versus 4.9 months (95% CI, 3.63–6.38) in the absence of TACE group (HR = 0.96, 95% CI 0.56–1.64; p = 0.875, **Figure 2C**). And the median OS was 14.9m (95% CI, 8.74–21.02); vs 13.7m(95% CI, 10.23–17.17) (HR = 1.07, 95% CI 0.56–2.05; p =0.833, **Figure 2D**). Furthermore, the survival outcome of combined TACE therapy in different treatment groups was analyzed in **Figure 3**.

**Table 3:** Response to treatment according to RECIST ver. 1.1.

Variable	PL ( n = 32 ) (%)	PR ( n = 29 ) (%)	P value
Complete response	1 (3.1)	0 (0)	
Partial response	3 (9.4)	3 (10.3)	
Stable disease	19 (59.4)	14 (48.28)	
Progressive disease	9 (28.1)	12 (41.38)	
Overall response rate	4 (12.5)	3 (10.3)	0.557
Disease control rate	23 (71.9)	17 (58.6)	0.207

RECIST, Response Evaluation Criteria in Solid Tumors.

### Treatment toxicities

The treatment-related AEs that occurred during the treatment with ICIs combined with lenvatinb or regorafenib therapy are described in **Table 4**. Of the 61 patients, 49 (80.3%) patients experienced any grade of AEs during treatment. Most patients during treatment experienced grade 1–2 TRAEs.

**Table 4:** Summary of adverse events.

Adverse event n (%)	PL group (n = 32)		PR group (n = 29)	
	Any grade	Grade ≥ 3	Any grade	Grade ≥ 3
Elevated AST level	3 (9.38)	0	4 (13.79)	0
Hyperbilirubinemia	2 (6.25)	0	3 (10.34)	0
Thrombocytopenia	5 (15.63)	0	5 (17.24)	2 (6.90)
Hypertension	12 (37. 5)	2 (6.25)	8 (27.59)	0
Diarrhea	6 (18.75)	0	4 (13.79)	0
Fatigue	8 (25)	0	7 (24.14)	0
Hoarseness	3 (9.38)	0	3 (10.34)	0
Proteinuria	4 (12.5)	2 (6.25)	0	0
Nausea	1 (3.13)	0	0	0
Hand-foot skin reaction	9 (28.13)	1 (3.13)	15 (51.72)	5 (17.24)
RCCEP	4 (12.5)	0	3 (10.34)	1 (3.45)
Rash	2 (6.25)	0	4 (13.79)	0
Hypothyroidism	6 (18.75)	0	7 (24.14)	0
Hyperthyroidism	5 (15.63)	0	4 (13.79)	0
ICIs-Induced hepatitis.	2 (6.25)	0	0	0
ICIs-Induced pneumonia	1 (3.13)	0	0	0

AST, aspartate aminotransferase; RCCEP, reactive cuntaneous capillary endotheial proliferation; ICIs, Immune Checkpoint Inhibitors.

In the PL group, the most common adverse events (≥Grade 3) were hypertension (n = 2,6.25%) and proteinuria (n = 2,6.25%). The most common adverse events (≥Grade 3) during PD-1 inhibitors plus regorafenib treatment were hand-foot skin reaction (HFSR) (n = 5,12.4%) and thrombocytopenia (n = 2, 6.90%)

### Prognostic factor analysis

The results of univariate and multivariate analyses of OS are listed in **Table 5**. Univariate analysis showed that independent risk factors for OS were ECOG PS (0-1versus2, HR = 5.16; 95% CI, 2.46-10.8; p < 0.001), Child-Pugh (B versus A, HR = 2.90; 95% CI, 1.29-6.54; p = 0.010), AFP level (≦̸400 versus >400 ng/ml, HR = 1.94; 95% CI, 0.99-3.30; p = 0.048). Furthermore, the independent risk factors for OS were ECOG PS (2 versus 0-1, HR = 0.223; 95% CI, 0.11-0.48; p < 0.001).

**Table 5:** Univariate and multivariate analysis of risk factors for overall survival.

Variables	Univariate analysis			Multivariate analysis		
	H.R	95% CI	P-value	H.R	95% CI	P-value
Gender (M vs F)	1.50	0.53-4.4	0.436			
Age (≥50 vs<50)	0.66	0.35-1.25	0.202			
ECOG PS(0-1vs 2)	5.16	2.46-10.8	<0.001	0.223	0.11-0.48	<0.001
BCLC (B vs C)	0.99	0.49-2.00	0.97			
Child-Pugh (A vs B)	2.90	1.29-6.54	0.010	0.57	0.25-1.33	0.192
HBV (yes vs no)	0.68	0.29-1.59	0.373			
Liver cirrhosis (yes vs no)	0.93	0.41-2.1	0.866			
Microvascular invasion (yes vs no)	1.11	0.62-2.0	0.727			
Extrahepatic spread (yes vs no)	1.16	0.59-2.28	0.668			
AFP (≥400μg/L vs <400μg/L)	1.94	0.99-3.30	0.048	0.65	0.33-1.29	0.218
Combination of TACE (yes vs no)	0.932	0.487-1.786	0.833			
Treatment option (PL vs PR)	0.9	0.45-1.79	0.764			

H.R, hazard ratio; CI, confidence interval; ECOG PS, Eastern Cooperative Oncology Group Performance status; AFP, alpha fetoprotein; BCLC, Barcellona Clinic Liver Cancer; HBV, hepatitis B virus; TACE, transarterial chemoembolization.

## Discussion

At present, sorafenib is still the first-line treatment of advanced HCC (15). However, the disadvantages of poor tumor control effect and relatively more toxicity are the most common cause of failure and mortality. Systemic therapy for second-line treatment of HCC has changed significantly (16), since the official approval of the molecular targeted agent (Regorafenib, Cabozantinib, Ramucirumab) and immune checkpoint inhibitors (Nivolumab and Pembrolizumab) (17, 18). But relevant studies show that their potential survival benefit is still very limited (ORR 9.2%~24.1%, PFS 3.1m~5.2m OS 8.5m~15m) (19, 20).According to the latesresearch results, ICIs combined with MTA can further improve the ORR、PFS and OS. After patients with advanced hepatocellular carcinoma were treated with atezolizumab plus bevacizumab, pembrolizumab combined with lenvatinib, pembrolizumab combined with regorafenib, nivolumab combined with lenvatinib and camrelizumab combined with apatinib, the ORR reached 33.2% ~ 54.2%,PFS 5.4m~8.6m and OS 20.3m~21.5m (21–24). Phase 1b study of lenvatinib/regorafenib plus pembrolizumab as first-line treatment for advanced HCC, which results showed that they were expected to produce better results (12, 22).And there are few data on ICIs combined with MTA in the second-line treatment of advanced HCC. Therefore, the purpose of this retrospective study was to investigate the efficacy and safety of PD-1 inhibitors plus lenvatinib and PD-1 inhibitors plus regorafenib in the treatment of advanced HCC patients who experienced disease progression during sorafenib treatment.

The clinical benefit found in the study may be attributed to the synergistic antitumor effect of PD-1 inhibitors and MTA, which can not only reshape the immune microenvironment (25, 26), but also promote the normalization of immune-competent cell functions (27–29).Lenvatinib, an inhibitor of VEGF receptors 1-3, FGF receptors 1-4, PDGF receptor α, RET, and KIT, which can normalize tumor blood vessels and destroy the hypoxic microenvironment of tumors, so as to enhance PD-1 checkpoint blocking of HCC (30, 31).Keynote-524 study (12)was conducted to explore pembrolizumab combined with lenvatinib as a first-line treatment for patients with advanced HCC. Combination treatment demonstrated that ORR of 36% and DCR of 88%, and mPFS of 9.7 months. Identically, studies also have shown that the combination of regorafenib and PD-1 inhibitors has synergistic effect, which can normalize tumor vasculature and induce sustained M1 polarization (32, 33). It plays immunosuppressive role of the tumor microenvironment (driven by regulatory T cells which may be targeted with anti-PD1) (34, 35). Safety signals and encouraging antitumor activity were also confirmed by clinical studies. The Phase Ib Trial (REGONIVO) demonstrated that the combination of regorafenib plus nivolumab had a manageable safety profile and encouraging antitumor activity in patients with gastric and colorectal cancer (36).The updated data (abstract No: 4078) published in the 2021 ASCO, Phase Ib study of regorafinib combined with Pembrolizumab in the first-line treatment of advanced HCC (NCT03347292) showed that ORR was 31% and DCR was 89%(7 had PR and 14 had SD), showing good antitumor activity and overall safe tolerance (22).

In this study, these analyses showed that using PD-1 inhibitors plus lenvatinib had better ORR and DCR than using PD-1 inhibitors plus regorafenib (ORR,12.5%vs. 10.3%, respectively, p = 0.557; DCR,71.9%vs. 58.6%, respectively; p < 0.207), but there was no statistical difference. However, median PFS and OS were not significantly different between the two groups in Kaplan-Meier survival analysis (PFS: 5.3 months vs 4.0 months, p = 0.512; OS: 14.1 months vs 13.7 months, p = 0.764 for the PL group vs PR group). Our finding is far worse than those in the RESCUE trial (37).

The study updated at the ASCO meeting in 2021, showed that camrelizumab combined with apatinib (selective VEGF 2 inhibitors) for second-line treatment of advanced HCC, with a median follow-up time of 29.1 months and an ORR of 22.5% (RECIST v1.1 standard), mOS was 21.8 months (17.3-26.8), and the 2-year OS rate was 44.6%. The patients included in RESCUE were only Child A, ECOG 0-1 score and macrovascular invasion (24.2%). However, the patients included in our study were relatively more late-staged with the majority of the patients in the PL/PR group [Child B (18.8%/10.3%), BCLC C (71.9%/79.3%), Extrahepatic spread (62.5%/79.3%)], reflecting real‐world data.

The author’s team believes that two combination regimes as the second-line therapy for patients with advanced HCC who failed sorafenib treatment can prolong survival time, compared with regorafenib or ICIs alone. The PL group seemed to have better tumor response and PFS compared with the PR group; however, their survival benefit of PL patients is not sustainable. In addition, patients in PR group have poor tolerance to regorafenib in clinical practice, which may be the latent reason for this result. In our study, it was found that the combination of locoregional treatment in the second-line treatment may not obtain survival benefit. These durations were different from the median PFS (7.4 months) and OS (14.3 months) of patients with HCC treated with regorafenib combined TACE for unresectable HCC with previous systematic treatment (38).The differences in the median PFS (4.2m vs 7.4m) outcomes between our study and previous study were likely due to differences in baseline patient characteristics. TACE combined with regorafenib included small proportions of patients with BCLC C (52.6%) or few patients with extrahepatic metastasis (34.2%), whereas our study included a larger proportion of patients with BCLC C (79.3%). Given the similar OS (14.9m vs 14.3m) associated with these combination therapies, patients with previous systematic treatment may not be ideal candidates for TACE plus systematic treatment as second line treatment. Although our outcome is currently negative, new advances in immunotherapy and locoregional therapy of HCC are paving the way for more powerful treatment strategies (39).

Treatment options using PD-1 inhibitors plus lenvatinib or regorafenib was not related to overall survival. Our study found that ECOG PS (0-1 vs 2) was an independent factor associated with mortality for patients with advanced HCC after sorafenib failure in multivariate analysis. The analyses showed that OS in the ECOG PS (0-1) group were significantly improved when compared with ECOG PS (2) group. (20.7m vs 6.8m, p<0.001). The author’s team believes that the basic physical state of patients is an important factor affecting the treatment benefits of patients in the practice of clinical practices. They have better tolerance to drugs and have the opportunity to receive locoregional treatment.

Overall, the adverse reactions incidence of two groups was similar to previous studies (12, 22).The study found that the PR group had significantly higher proportions of TRAEs than the PL group (79.3% vs 62.5%, p<0.001), with the leading four adverse events being HFSR (51.72%), hypertension (27.59%), fatigue (24.14%) and hypothyroidism (24.14%). Moreover, eight patients (10.2%) had severe TRAEs requiring permanent cessation of treatment, including five with HFSR, two with thrombocytopenia and one with RCEEP. Compared with PR group, the PL group had lower incidence of TRAEs during treatment, with hypertension (37. 5%), HFSR (28.13%) and hypothyroidism (18.75%). Only five patients (15.6%) had severe TRAE over grade 3, and presented with hypertension (n = 2,6.25%), proteinuria (n = 2,6.25%), and HFSR (3.13%). Although using PD-1 inhibitors plus regorafenib has more TRAE and poorer life quality, the long-term survival benefits appear equivalent to PR and PL in medical practices.

This article has several limitations. First of all, this is a retrospective study and the patients come from one medical center and may be affected by specific treatment practices. Secondly, the baselines of different ICIs groups are not balanced, and unable to perform subgroup analysis of immune checkpoint inhibitors used by patients. In the follow-up study, we will further explore the relationship between different types of immune checkpoint inhibitors and patient survival outcomes. Thirdly, about half of the enrolled patients using TACE treatment, and there was a certain bias in the evaluation of curative effect. Finally, the analysis of PFS or OS show no notable statistical significance due to the small sample size of the patients.

In conclusion, our results show that the combination of PD-1 inhibitors plus lenvantinib or regorafenib in the treatment of advanced HCC has acceptable survival benefits and controllable side effects, however, their PFS and OS did not differ significantly. Further research is needed on whether advanced HCC patients are combined with local treatment as second-line treatment. Multi-center, prospective, randomized controlled trials and large samples studies are needed to explore more economical and effective treatment regime in the future.

## Data availability statement

The raw data supporting the conclusions of this article will be made available by the authors, without undue reservation.

## Ethics statement

The studies involving human participants were reviewed and approved by The ethics committee of the Second Affiliated Hospital of Nanchang University. The patients/participants provided their written informed consent to participate in this study.

## Author contributions

YX, KS and YM conceived the study. YX and SF conducted the work. YX, KS and JZ obtaining and analyzed the data. YX wrote the manuscript. YM reviewed the manuscript. All the authors listed have read and approved the manuscript. All authors contributed to the article and approved the submitted version.

## Funding

This project was financially supported by the grants from the Natural Science Foundation of China (82160602).

## Conflict of interest

The authors declare that the research was conducted in the absence of any commercial or financial relationships that could be construed as a potential conflict of interest.

## Publisher’s note

All claims expressed in this article are solely those of the authors and do not necessarily represent those of their affiliated organizations, or those of the publisher, the editors and the reviewers. Any product that may be evaluated in this article, or claim that may be made by its manufacturer, is not guaranteed or endorsed by the publisher.
